# Prevalence and Reasons for Tooth Loss in a Sample from a Dental Clinic in Brazil

**DOI:** 10.1155/2012/719750

**Published:** 2012-08-29

**Authors:** Andréia Montandon, Elizangela Zuza, Benedicto Egbert Toledo

**Affiliations:** ^1^Department of Social Dentistry, São Paulo State University (UNESP), Rua Humaitá, 1680, 14801-903 Araraquara, SP, Brazil; ^2^Department of Master of Dental Sciences, Educational Foundation of Barretos (UNIFEB), Avenida Prof. Roberto Frade Monte 389, 14783-226 Barretos, SP, Brazil

## Abstract

*Purpose.* To evaluate the prevalence and reasons for teeth extractions in a sample from a dental clinic in Brazil. *Methods.* The prevalence of teeth mortality was analyzed by gender, age, tooth type and reasons for extraction on 800 teeth of 439 subjects, whose data was collected in clinical records in a convenience sample. *Results.* The groups with range from 35 to 44 years, 45 to 54 years and 55 to 64 years revealed significantly greater number of teeth extractions than other age groups (*P* < 0.0001). The anterior teeth loss increased significantly with aging, while the tooth mortality of premolar and molar were higher in younger people. The caries was the more prevalent reason for tooth mortality among young and adults up to 44 years old, while the periodontal disease was the main reason for extractions from 45 years old until range of 81 years (*P* < 0.0001). *Conclusions.* It can be suggested that some reasons for tooth loss were age-dependent, but the caries and the periodontal diseases were the main reasons for tooth mortality in this Brazilian sample.

## 1. Introduction 

Studies concerning the epidemiology in dentistry have showed that dental caries and periodontal diseases are the most prevalent pathologies that affect the oral cavity. Previous studies performed by American researchers had suggested that dental caries was the main reason for teeth extraction, and other studies accomplished in New Zealand, Sweden, and even in Brazil confirmed that caries may lead to tooth mortality [1–5]. On the other hand, some studies suggested that periodontal disease was the most prevalent reason that leads to tooth loss. Thus, controversial findings could be explained by differences in the characteristics of the study population, immunological and genetic factors, cultural beliefs, and socioeconomic characteristics, among others. Immunological and genetic reasons are some of the contributory factors that may explain why some populations exposed to the same bacterial etiologic factors did not develop similar pathological conditions [6, 7].

 Populations with poorer socioeconomic conditions have shown higher prevalence and extent of teeth mortality, which increases with aging [8–10]. The prevalence, extent, and risk indicators for tooth loss were studied in a representative Brazilian population, showing that 94% of the subjects had experienced tooth loss [8]. The authors verified a tooth mortality mean of 11.2, ranging from 5.5 to 20.2 teeth in subjects from 30 to 39 years old and 60+ years age groups, respectively; gender, socioeconomic status, cigarette smoking, caries experience, and attachment loss were important risk indicators. 

On the other hand, a decline in the tooth loss can be verified in developed countries in the last years [7, 11, 12], which can be explained by preventive programs and higher accessibility to the oral health care that have been decreasing the extractions [13, 14]. Brazil is a country in development progress (underdeveloped), and some studies verified the prevalence of tooth extractions in Brazilians [4, 8–10, 15, 16], but few of them have verified the reasons for the tooth mortality in this population. Thus, the aim of this study was to describe the epidemiological data of the prevalence and reasons for teeth extractions in a convenience sample of Brazilians.

## 2. Materials and Methods

### 2.1. Sample Selection

This study was approved by the Ethics Committee in Human of Araraquara Dental School (UNESP—process number 45/99). A review of 800 clinical records was performed to select 439 patients with tooth loss from 1999 up to 2002 in Dental Clinics at State University of São Paulo (UNESP), Araraquara city, Brazil (Sudeste region). From the 800 clinical records, 361 subjects were excluded by X-ray absence or absence of specifications of the reasons for tooth mortality, and 439 patients were enrolled in this study. From these 439 patients, 800 teeth with X-ray and specifications of reasons for tooth loss were selected.

### 2.2. Data Collection

The clinical records were evaluated considering the gender and the age groups. Additionally, the prevalence of tooth loss was evaluated by reasons of extractions and type of teeth (molars, premolars, incisors and canines). The incisors, and canines were nominated only as anterior teeth. The reasons for teeth mortality were adapted based on Cahen et al. [17] as follows: (1) caries: it is properly caries; (2) endodontic problems, such as pulp inflammation, necrosis, or even tooth fracture; (3) periodontal diseases: tooth extraction by periodontal attachment loss; (4) eruption problems: tooth loss by dental impacation; (5) prosthetics: indication for extraction by prosthetic reasons; (6) trauma: extractions by external trauma; (7) orthodontics: tooth extraction by orthodontic indications; (8) occlusal problems: extractions by occlusal dysfunction, such as extrusion; (9) other reasons: any other determined reasons, such as iatrogenic factors. A single examiner accomplished the clinical records assessment, and data was described in a proper form. 

### 2.3. Statistical Analysis

Descriptive data were expressed by means of numbers and percentages. Chi-square statistics (*χ* ^2^) were applied, considering *P* < 0.05 as a statistical significant difference. Adjusted residual chi-square (*χ* ^2^) was calculated, considering 1.96 (*α* = 0.05) and 2.573 (*α* = 0.01).

## 3. Results

Table 1 shows the data collected from 1999 to 2002, the number of subjects, and teeth included in this study. The number of subjects according to age groups and gender ratio can be verified in Table 2, that shows a similar prevalence of tooth loss between gender in all age groups (*P* = 0.67).  Table 3 shows that the groups with range from 35 to 44 years, 45 to 54 years, and 55 to 64 years revealed significantly greater number of teeth extractions than other age groups (*P* < 0.0001). The anterior teeth loss increased with aging and the extractions of premolars and molars decreased with aging, while the tooth mortality of premolar and molar were higher in younger people (Table 4). The results showed that caries and periodontal diseases were statistically significant reasons for tooth loss when compared to the other factors (*P* < 0.0001; Table 5).  Table 6 shows that caries was the most prevalent reason for tooth mortality among young and adults up to 44 years old, while the periodontal disease was the main reason for extractions from 45 years old until range of 81 years. Other reasons for teeth extractions can be verified as follows: iatrogenic factors (9.9), eruption problems (6.4), orthodontics (5.7), prosthetic indication (3.6), trauma (2.6), and occlusion problems (1.1). It can be seen in Table 6 that tooth mortality for some reasons such as orthodontic, occlusal problems, and other reasons was significantly higher (*P* < 0.0001) among younger, and by prosthetic reasons was significantly higher in older people (*P* < 0.0001).

## 4. Discussion

Our data showed that the number of male and female subjects with tooth loss was similar among all age groups. Conversely, the study by Barbato et al. [10] verified higher prevalence of tooth mortality in women than in men. Other studies assessed the tooth mortality among the urban and rural adult population of Dharwad district (India) in 1223 subjects (685 urban and 538 rural) and found that females compared to males had higher tooth loss [18]. Moreover, our findings showed that the age group between 35 to 44 years old presented a mean tooth loss of 2.3, which was lower than the mean of 11 verified in the other study [10]. This difference could be explained by the sample size of the studies, which was of 439 Brazilians in our sample and a major population of 13.431 Brazilians in the study by Barbato et al. [10].

Our findings showed that among adolescents aged from 15 to 19 years, the prevalence of tooth extraction was 8.0%, while in a greater population of 16.833 Brazilian adolescents it was 38.9% [19]. Probably this difference could also be related to the sample size of the studies that is too discrepant. The results in the present study demonstrated higher missing teeth for premolars (43.1 to 51.6%) and molars (48.4 to 51.0%) among the youngest subjects (15 to 24 years), and the dental caries was the main reason for teeth extractions (67.2%). Our findings are in agreement with previous studies that have demonstrated that the first molars were the most prevalent missing teeth in Brazilian younger people [9, 15, 19]. Barbato and Peres [19] found more than 55% of missing first molars, and dental caries experiences were found in 92.71% of all teeth lost. Other studies also suggested that the first molars were the most frequently missing teeth in subjects aged from 14 to 29 [9] and from 13 to 16 years [15]. 

The caries (38.4%) and periodontal disease (32.3%) were the most prevalent conditions for tooth mortality in the present study with similar prevalence. Conversely, other study on Brazilians suggested that the main reason for tooth extraction was the caries (70.3%) and not the periodontal disease (15.1%), but this study showed findings of a small sample with 404 teeth extracted [16]. In a Caribbean population—Antigua, Viagnarajah [20] also suggested that caries was the main reason for tooth extraction (61.6%), and the periodontal disease was the next more frequent reason for tooth loss (29.9%). 

Our results showed that periodontal disease was more prevalent among older subjects over 40 years of age. Some studies are in agreement with our findings, showing that periodontitis became the most prevalent reason for tooth mortality in people near 40 years old [10, 20–26]. In addition, other reasons for teeth extractions were found in the present study as follows: iatrogenic factors (9.9%), eruption problems (6.4%), orthodontics (5.7%), prosthetic indication (3.6%), trauma (2.6%), and occlusal problems (1.1%). Caldas [16] also evaluated other reasons for extractions in 404 teeth, such as prosthetic reasons (6.4%), third molar-eruption disorders (3.7%), orthodontic reasons (2.5%), and trauma or patient′s request (1%), which showed lower prevalence than the caries and periodontal disease.

 Considering that few studies evaluated reasons for teeth losses in Brazilians, our retrospective study may be useful in clinical dentistry and used as baseline data regarding prevalence and reasons for teeth extractions in other populations; moreover, other prospective studies might be performed. 

## 5. Conclusions

Within the limits of this study, it can be suggested that some reasons for tooth loss were age dependent, but the caries and the periodontal diseases were the main reasons for tooth mortality in this Brazilian sample.

## Figures and Tables

**Table 1:** Data collected for the study in different years.

Years	Subjects (number)	Teeth (number)	Mean number of teeth *per* subject
1999	138	274	2.0
2000	151	239	1.6
2001	127	235	1.9
2002	23	52	2.3

Total	439	800	1.8

**Table 2:** Number of subjects according to age groups and gender ratio (*n* = 439).

Age group (years)	Gender* (male/female)	Total
15–19	15/16	31
20–24	12/15	27
25–29	13/10	23
30–34	21/17	38
35–44	34/24	58
45–54	45/32	77
55–64	55/39	94
65–74	28/31	59
75–84	14/18	32

*P* value	0.67	—

*Chi-square test for categorical data in different age groups (*P* value > 0.0.5 does not indicate statistically significant difference).

**Table 3:** Number and percentage (%) of teeth extractions by age group.

Age group	Number of teeth	% of teeth extracted	Mean number of
			teeth extracted
15–19	64	8.0	2.1
20–24	51	6.4	1.9
25–29	24	3.0	1.0
30–34	45	5.6	1.2
35–44*	131	16.4	2.3
45–54*	163	20.4	2.2
55–64*	180	22.5	1.9
65–74	105	13.1	1.8
75–84	37	4.6	1.2

15–81	800	100.0	1.8

Chi-square test (*χ* ^2^) = *P* < 0.0001. *> 2.576 (*α* = 0.01) adjusted residual chi-square.

**Table 4:** Prevalence (%) of extractions by age group and type of teeth.

Age group	Tooth			
	Anterior	Premolar	Molar	Total
	*N* (%)	*N* (%)	*N* (%)	*N*
15–19	—	33 (51.6)*	31 (48.4)*	64
20–24	3 (5.9)	22 (43.1)	26 (51.0)*	51
25–29	5 (20.8)	8 (33.3)	11 (45.9)	24
30–34	11 (24.4)	14 (31.1)	20 (44.5)	45
35–44	33 (25.2)	42 (32.1)	56 (42.7)*	131
45–54	62 (38.0)	54 (33.2)	47 (28.8)	163
55–64	84 (46.7)*	52 (28.9)	44 (24.4)	180
65–74	69 (65.7)*	25 (23.8)	11 (10.5)	105
75–84	25 (67.6)*	7 (18.9)	5 (13.5)	37

**15–81**	292 (36.5)	257 (32.1)	251 (31.4)	800

Chi-square test (*χ* ^2^) = *P* < 0.0001. * > 2.576 (*α* = 0.01) adjusted residual chi-square.

**Table 5:** Reasons for teeth extractions (*N*: number; % percentage).

Reasons	Frequency	
	*N*	%
Caries	307*	38.4
Periodontal diseases	258*	32.3
Eruption problems	51	6.4
Prosthetics	29	3.6
Trauma	21	2.6
Orthodontics	46	5.7
Occlusal problems	9	1.1
Others reasons— iatrogenic and accidental	79	9.9

Total	800	100.0

Chi-square test (*χ* ^2^) = *P* < 0.0001. * > 2.576 (*α* = 0.01) adjusted residual chi-square.

**Table 6:** Reasons for tooth extraction by age groups.

Age group					Reasons				
	C	PD	E	P	T	O	OP	Others	Total
	*N* (%)	*N* (%)	*N* (%)	*N* (%)	*N* (%)	*N* (%)	*N* (%)	*N* (%)	
15–19	43	—	18	—	—	—	—	3	64
	(67.2)*		(28.1)*					(4.7)	

20–24	18	—	24	—	—	4	—	5	51
	(35.3)		(47.1)*			(7.8)		(9.8)	

25–29	12	—	5	—	—	—	—	7	24
	(50.0)		(20.8)*					(29.2)*	

30–34	13	6	2	—	2	8	4	10	45
	(28.9)	(13.3)	(4.4)		(4.4)	(17.8)*	(8.9)*	(22.2)*	

35–44	73	34	—	—	2	—	2	20	131
	(55.7)*	(26.0)			(1.5)		(1.5)	(15.3)*	

45–54	57	63	—	7	8	10	—	18	163
	(35.0)	(38.7)*		(4.3)	(4.9)	(6.1)		(11.0)	

55–64	56	83	2	8	6	13	3	9	180
	(31.1)	(46.1)*	(1.1)	(4.5)	(3.3)	(7.2)	(1.7)	(5.0)	

65–74	23	54	—	10	3	11	—	4	105
	(21.9)	(51.4)*		(9.5)*	(2.9)	(10.5)		(3.8)	

75–84	12	18	—	4	—	—	—	3	37
	(32.4)	(48.7)*		(10.8)				(8.1)	

**15–81**	**38.4**	**32.3**	**6.4**	**3.6**	**2.6**	**5.7**	**1.1**	**9.9**	**800**

C: caries; PD: periodontal disease; E: eruption problems; P: prosthetics; T: trauma; O: orthodontics; OP: occlusal problems; Others: other reasons (accidents or iatrogenic factors). Chi-square test (*χ* ^2^) = *P* < 0.0001. *> 2.576 (*α* = 0.01) adjusted residual chi-square.
